# Some Points in the Treatment of Dyspepsia

**Published:** 1891-11-21

**Authors:** 


### THE PRACTITIONER'S MIRROR AND RETROSPECT.

*[The Editor will be glad to receive offers of co-operation and contributions from members of the profession. All letters should be addressed to* THE EDITOR, THE LODGE, PORCHESTER SQUARE, LONDON, W.]

### MEDICINE.

### SOME POINTS IN THE TREATMENT OF DYSPEPSIA.

One of the chief difficulties in the treatment of dyspepsia depends on the number of causes giving rise to symptoms which are grouped together under the name of dyspepsia; and, in fact, on the number of functions which the stomach and the intestinal tract have to perform, any one of which may be deranged. There is the peristaltic action, the secretion of lactic and hydrochloric acids and other juices, the absorption of peptones, and other products, the induction of processes of fermentation and the stopping of the action of introduced bacteria, adaptation of the machinery to varying and irritating food and to irregular meals, some form of respiration through the walls, and of excretion of various poisons into the intestinal tract. A stoppage of any one of them quickly throws the whole machine out of gear. Yet we are apt to forget the existence of nine-tenths of these functions, and certainly find it hard to distinguish their derangements. How important the least of them may be was shown recently in some experiments on the effect of injections of morphine. It was proved that morphine when introduced by hypodermic injection made its appearance shortly after in the stomach of the animal. This excretion into the stomach and re-absorption was so great that, if the stomach was washed out several times soon after the hypodermic injection, a very considerable proportion of the morphia could actually be recovered from the stomach and removed. The discoverers laid stress on the importance of the fact as affording a method of saving life in cases of an overdose, for they considered that the chief effects of the drug on the nervous system followed its reabsorption from the stomach; but the importance with regard to digestion of the excretion of similar poisons is even greater. It has long been known that one function of the liver is to catch various poisons and return them with the bile into the intestines, but no one has investigated thoroughly the condition of excretion into the stomach, though there is reason to think that many poisonous matters are, during the course of diseases, thus excreted. Here we see one reason for the immense importance of purgatives in many gastric and intestinal disorders, and the reputation which they have long held from their power of carrying off effete matters before they can be reabsorbed, or accumulate so as to irritate the mucous membrane and interfere with digestion. Passing over the black vomit of yellow fever, for which such an origin has recently been claimed, we find the stomach frequently irritated by the matters excreted into it in Bright's disease, and the gastric irritation in acute gout is probably owing in part to the local effects of uric acid. In short, in numerous complaints we find irritant matters poured out into the stomach which are the causes of dyspepsia, so that the organ is, to use a common phrase, more sinned against than sinning. The indication usually is to treat the source of the primary disease. Secondary dyspepsia is of course commonly recognised in many cases, such as when arising from venous congestion in heart complaints, but the point we would emphasise is that many apparently simple dyspepsias may depend on some general state, such as lithæmia, or a faulty action of the kidney or lungs. Thus it is often a difficult question to decide whether a sick-headache is the result of irregular nerve discharges or a poison circulating in the blood, or some irritant either absorbed from or excreted into the stomach. Hydrochloric acid may relieve this either by aiding digestion and antisepsis, which are more perfect when it is present, or, as Dr. Haigh thinks, by driving uric acid out of the circulation.

One most fruitful source of dyspeptic troubles is found in the entrance of many kinds of bacteria and ptomaines. In health, when the gastric juice is normal, and contains a due proportion of free acid, it has a powerful germicidal effect, but with a slight alteration in composition or the time of secretion, or from retarded peristalsis, or from irregular or excessive meals, this action may fail, and the stomach and digestive tract becomes the scene of endless abnormal fermentations. These interfere with the normal ones. An excessive or long-continued lactic acid process, for instance, interferes with the hydrochloric acid one, and irritation of the mucous membranes follows. These septic matters may be thus a cause or an effect of the dyspepsia, but in either case they must be got rid of before the condition of the mucous tissues can be improved. Washing out the stomach, or some other method of antisepsis is often, therefore, a primary indication.

Nature, of course, carries out this aim by purgation, vomiting, and the action of the gastric and biliary secretions, and we find in practice that better results are often got by aiding her with moderate doses of, say, calomel, or from a complete change of diet than from the administration of naphthol and other antiseptics. With respect to a change of diet, Dr. Hamilton has pointed out that much dyspepsia depends on a hyperdigestion. The proteids may pass beyond peptones, starch may pass not only into sugar but into additional fermentations. If, therefore, our dyspepsia arises from the hyperdigestion of one type of food, such as starch, and we feed the patient entirely for some days on proteids and gelatines, a natural and very rapid improvement takes place.

This has long been recognised in the management of infants, and if we fail to get milk digested, a common method is to put the child entirely on a veal broth diet for some days. The plan is rarely tried with adults. To be effective, such a change must entirely exclude for a time either all flesh food or all starches, according to the element with which difficulty occurs. In fact many patients can be best treated by periodical fasting days, which consist not in a so-called light diet of various kinds, but in a purely carbo-hydrate or proteid aliment. After a rest of this kind the digestion will in many cases regain its power and the excess of lactic acid, butyric, and other bye products will disappear. Though we are all familiar with the importance of a change of diet, a thorough change like this is almost unknown. We give mutton for beef, cocoa for tea, picking out articles which are mysteriously called "light," without reference to physiology, till the patient worries himself into believing that nine-tenths of his ordinary food is injurious. He cuts off one article after another, reduces himself to semi-starvation, and finds no relief but in artificially digested foods and constant drugs. In fact dieting when done by rule of thumb is perhaps even more abused than drugs are.

In the common chronic types of dyspepsia where there is neither local organic disease, nor the action of the poison of a general disease, nor any specially irritating or poisonous food, we have to contend with the frequently recurring failure of the stomach to do its work regularly and painlessly. This may depend either on deficient peristalsis and other nervous defects, or on abnormal secretions. If the food is not passed on in proper time, it may act in many cases as an irritant, causing abnormal secretion of the gastric juice. Hence the value of nerve tonics, such as nux vomica, massage, moderately active exercise and hot liquids towards the end of digestion; and the ill effects of violent emotion in stopping peristaltic action. In short, the action of the organ is affected by much the same causes as that of the uterus in parturition, though normally unattended with painful sensations. The dyspepsias which depend on failure of proper secretion are for the most part bound up with those from other causes. However, the membrane easily takes on a catarrhal condition and becomes either too irritable or too sluggish. In the latter case, if peristalsis is active, the food is soon handed over from the gastric to the pancreatic juices, and probably finds some part of the machinery active. The administration of hydrochloric acid, especially in older people and when the tongue is pointed and surrounded by a red margin, seems to give relief; but if peristalsis is also deficient there is soon developed irritation and bacterial changes. In the cases with pale flabby tongue, distension, and moderate pain, and acid reactions, the question of giving alkalies as the most commonly useful remedies is important.

Roberts has recently pronounced in favour of the persistent use of the sodamint tablets and similar preparations if taken in moderation and at proper times. This question of the time is crucial. A patient receives a draught, with soda, chloroform, sal-volatile, and a bitter, and takes it at perhaps ten o'clock, three o'clock, and seven o'clock. That is, quite irrespective of the stage of digestion, and naturally finds himself no better. Yet such a draught, or a piece of toast or fluid peptone taken at the beginning of each meal would have stimulated and regulated digestion. If taken in the middle of the acid tide some 2½ hours later, it would perhaps have allayed some irritation, but it would certainly stop the digestion altogether, and in many cases leave a mass in the stomach to be expelled by vomiting. A cup of hot liquid instead or exercise would towards the end of gastric digestion complete the process, with possibly a few grains of soda afterwards, if pain continues when the stomach is empty.

By care in giving once or twice a day an alkaline and sedative draught with meals only, and employing exercise, onics, and other means for increasing peristalsis, and by occasionally taking a complete fast from either proteid or carbohydrate food, many attacks of gastric troubles can be brought to an end. There are patients who seem comfortable only when they take an alkali daily, and go on for long periods in this way in apparently perfect health. In these cases it becomes a question whether there is not some lithæmic condition which the alkali neutralizes, and which would otherwise show itself first by dyspepsia. We are only just beginning to learn a little of the true processes of digestion, and there is certainly no subject with which the comfort and happiness of mankind is more closely connected.

